# Anxiety and Avoidance in Adults and Childhood Trauma Are Associated with Negative Religious Coping

**DOI:** 10.3390/ijerph17145147

**Published:** 2020-07-16

**Authors:** Alice Kosarkova, Klara Malinakova, Jitse P. van Dijk, Peter Tavel

**Affiliations:** 1 Olomouc University Social Health Institute, Palacky University Olomouc, 771 11 Olomouc, Czech Republic; klara.malinakova@oushi.upol.cz (K.M.); j.p.van.dijk@umcg.nl (J.P.v.D.); peter.tavel@oushi.upol.cz (P.T.); 2 Department of Community and Occupational Medicine, University Medical Center Groningen, University of Groningen, 9713 AV Groningen, The Netherlands; 3 Graduate School Kosice Institute for Society and Health, P.J. Safarik University in Kosice, 040 11 Kosice, Slovakia

**Keywords:** negative religious coping, childhood trauma, attachment anxiety, attachment avoidance

## Abstract

Religion as a coping strategy is mostly connected with positive health outcomes. Yet, negative religious coping (NRC) has been associated with rather negative outcomes that affect one’s health. The aim of this study was to explore whether insecure adult attachment and childhood trauma are associated with higher NRC. A sample of Czech adults (*n* = 531, 51.1 ± 17.2 years; 43.5% men) participated in a survey. As measures, the NRC subscale of the Brief RCOPE, the Experiences in Close Relationships-Revised questionnaire, and the Childhood Trauma Questionnaire-Short Form (CTQ-SF) were used. From the whole sample, 23.7% respondents reported higher NRC. Respondents with higher anxiety in close relationships were more likely to use negative coping strategies, with an odds ratios (OR) of 1.27 (95% confidence interval 1.01–1.59). Similarly, avoidance was associated with negative coping OR = 1.41 (1.13–1.75). Moreover, each subscale of the CTQ-SF revealed a significant association with high summary NRC. Respondents who reported physical neglect scored highest on summary NRC with OR = 1.50 (1.23–1.83) after controlling for sociodemographic variables, but also for anxiety and depression. Our findings support the idea that childhood trauma experience and adult attachment style are associated with higher use of NRC strategies.

## 1. Introduction

Religion belongs among well-documented coping strategies, through which one can understand and deal with stressors [1]. When assessing religious coping, two forms can be distinguished: positive religious coping (PRC) and negative religious coping (NRC) [2]. PRC strategies reflect a secure relationship with God, spiritual connectedness, and meaning in life. On the contrary, NRC is characterized by spiritual tension, and conflicts and struggles with God and others in one’s religious community [3].

As a multidimensional construct, religious coping has both positive and negative associations with health [2]. PRC has been associated with increased physical [4] and mental health [5], lower levels of depression [6], and a higher quality of life [7] compared with people who used NRC strategies. Regarding NRC, researchers reported mostly negative health outcomes and poorer psychological adjustment [3,6]. NRC strategies were associated with higher levels of depression [5,8], somatization or disordered eating pathology [5,9], worse quality of life, and lower life satisfaction [8,10] than in people using PRC strategies. Similarly, NRC strategies predicted worse physical functioning [11] and a decline in health [12,13], and were significantly associated with lower comprehension of one’s illness and distrust of treatment efficacy [10]. These strategies were also related to higher suicidal risk [10,14] and a higher risk of mortality [15]. Minimizing the negative outcomes of NRC is thus very vital. Therefore, it is important to understand why individuals use NRC.

The first reason people use NRC may lie in their attachment strategies. One’s beliefs about and relationship towards God have been found to be similar to human attachment relationships [16,17]. For example, avoidant attachment to a person was positively associated with avoidant attachment to God [17,18] and the desire to keep God at a distance [19,20]. Similarly, anxious attachment to a person was associated with anxiety in attachment to God [17] and thus may be related to a tendency to feel abandoned by God or church and even feel punished by God [19]. 

The second explanation could be that the inclination to draw on PRC or NRC strategies in crises could be associated with one’s image of God [21]. Whereas individual’s God concept (i.e., explicit image) can be influenced by many factors, including family, religious community or education and is usually expressed in verbal descriptions of God [22,23], one’s implicit image of God may be seen as the way one interacts with God at an emotional, relational and nonverbal level [24]. The development of the God image is closely connected to the attachment theory and relationship with a caregiver and thus one’s image of God might be strongly affected by childhood trauma, the experience of maltreatment, or insecure attachment to parents during childhood [25,26]. Many childhood abuse victims tend to view God in rather negative terms, such as unloving, distant, or controlling [26,27]. Victims of traumatic events also reported a negative impact on their religiosity [26]. Nevertheless, in some cases, different traumas were found to be related to an increase in spirituality, because of a person’s effort to understand why this had happened [28,29].

As a robust predictor of poor health-related outcomes, NRC has been separately assessed in some studies [9,30,31]. According to these studies, the prevalence of NRC varies from 7 to 50% in various populations [30]. This variation might be explained by the variability of criteria employed to determine the presence of NRC [32,33,34]. Other explanations could involve differences in the cultural context and situational or clinical factors. Thus far, most studies on religious coping and its associations with adult attachment or childhood trauma have been conducted outside of Europe [17,19,26,28,35]. Few studies have been carried out within a European context [36,37,38]. Thus, this study from the Czech Republic, which according to the Pew Research Centre [39] is the country with the highest percentage of religiously unaffiliated people in the world, could contribute to studies on NRC in very secular countries.

Therefore, the aim of this study is to explore the association of adult attachment and childhood trauma with NRC in a highly secular environment. We wanted to assess NRC, using both a total score and a more detailed analysis of individual items, to see which of these items showed the strongest association with our observed variables.

## 2. Materials and Methods

### 2.1. Participants and Procedure

The sample in our research was created by selecting from the original representative sample only the respondents who identified themselves as religious. The original sample of the Czech population aged fifteen years and older was obtained by using a two-step procedure. In the first step, the questionnaire and all further procedures were piloted among 206 participants. This led to the final version of the survey. In the second step, another 2184 participants were randomly chosen with the help of quota sampling and asked to participate in a study on health, life experiences, attitudes, and lifestyle. Quota sampling is a technique often used in research to imitate the known characteristics of the population in the sample, allowing relationships between subgroups to be observed. In this case the criteria that allowed the construction of a representative sample corresponding to the adult Czech population were used. Of these respondents, 384 (17.6%) refused to participate mainly due to their lack of time or no interest in the topic. The remaining sample consisted of 1800 respondents. Among whom only some reported themselves as religious; therefore, the final sample consisted of 531 participants.

Data was collected by professionally trained administrators in September and October 2016 during a standardized face-to face interview with the respondents. Participation in the survey was anonymous and voluntary and respondents did not receive compensation for their participation in the survey. Participants signed an informed consent form prior to the study; this stressed the possibility of leaving the study at any time without giving reasons. The study design was approved by the Ethics Committee of the Olomouc University Social Health Institute, Palacky University in Olomouc (No. 03/2016).

### 2.2. Measures

All instruments were available in the Czech language. Religious background was obtained using self-developed questions on religiosity: ‘At present, would you call yourself a believer?’ with possible answers: yes, I am a member of a church or religious society; yes, but I am not a member of a church or religious society; no; no, I am a convinced atheist. The question assessed whether respondents consider themselves religious and whether they are affiliated to a specific religion or religious practice.

Religious attendance was measured as the frequency of attending church or religious sessions using the question: “How often do you go to church or to religious sessions?” Possible answers were: never, occasionally; often, but not every week; once a week; more than once a week. Those who reported attending religious sessions at least once a week were considered attending. 

Prayer frequency was assessed by the question: “How much time do you devote to personal prayer (excluding religious gatherings)?” with possible answers: at least half an hour a day; approximately 10 min every day; approximately 10 min together per week; I pray only occasionally, I don’t pray.

Religious coping was assessed using the negative religious coping subscale (NRC) of the Brief RCOPE [3]. It is composed of 7 items rated on a seven-point scale with possible answers ranging from ‘not at all’ (1) to ‘a great deal’ (4) and the total score ranges from 7 to 28. NRC items reflect a religious struggle that grows out of a more tenuous relationship with God. In the analyses, NRC was assessed as a dependent variable. For the purpose of dichotomisation, the approach of Fitchett et al. [32] was followed for the further categorization of responses. Each of the item scores was dichotomized. Scores of 1 or 2 were recoded to ‘0’ (did not use NRC) and scores of 3 or 4 recoded to ‘1’ (used NRC). To determine the NRC sum, a dichotomous variable was created with a value of ‘1’ if any of the seven NRC items had a value of ‘1’ [30]. Cronbach’s alpha was 0.84 in our sample.

Experience in close relationships was assessed using the shortened version of the Experiences in Close Relationships-Revised (ECR-R-16) questionnaire [40], which was validated for the Czech environment [41]. It is composed of 16 items rated on a seven-point scale, with possible answers ranging from ‘totally disagree’ (1) to ‘totally agree’ (7), and measures two dimensions of attachment-related experience. Each subscale consists of eight items. The Anxiety subscale measures the extent to which people are insecure about the availability and responsiveness of a partner or a close relation, while the Avoidance subscale measures the extent to which people feel uncomfortable being close to others. In the main analyses, both subscales were assessed as a binary variable created by dichotomizing the score with the subscale’s upper quartile as the cut-off point. Cronbach’s alpha was 0.70 in our sample for both subscales.

To assess childhood trauma, the Childhood Trauma Questionnaire-Short Form (CTQ-SF) [42] was used. It is a standardized 28-item self-report inventory developed to measure the severity of five types of abuse and neglect in childhood or adolescence by the following subscales: Emotional Abuse, Physical Abuse, Sexual Abuse, Emotional Neglect, and Physical Neglect. Each subscale contains five items with a 5-point Likert-type scale ranging from ‘never’ (1) to ‘very often’ (5), leading to scores from 5 to 25 for each subscale. Besides these, the CTQ-SF also has a three-item minimisation/denial validity scale that was developed to detect the underreporting of maltreatment [42]. The CTQ-SF measure was introduced by the statement “The following questions are related to some of your childhood or adolescent experiences” in order to be sure that the trauma occurred in childhood/adolescence. Cronbach’s alpha for the CTQ-SF subscales in our sample ranges from 0.62 to 0.89.

Anxiety and depression were assessed by Anxiety and Depression subscales of the Brief Symptom Inventory (BSI-53) [43,44]. The introductory instruction was: “How much has the following symptoms problem distressed or bothered you during the past month?” It was followed by items rated on a five-point scale with possible answers ranging from “not at all” (0) to “extremely” (4). In the main analyses, both subscales dimensions were assessed as binary variables created by dichotomizing the score into the subscale’s upper quartile or below. Cronbach’s alpha for the Anxiety subscale was 0.83 and 0.88 for the Depression subscale. Gender, age, education, and marital status data were obtained through the questionnaire.

### 2.3. Statistical Analyses

In the first step, we described the background characteristics of the sample and the distribution of NRC item responses. Nonparametric methods were used to compare different sociodemographic groups. The Wilcoxon sign-rank test was used to compare gender; in other cases, when more than two groups were compared, we used the Kruskal-Wallis test. We then assessed the associations of two attachment dimensions, anxiety and avoidance, and the five types of childhood trauma experiences with negative religious coping (in total and each of the seven items separately) using a binary logistic regression model that was crude at first (Model 1), adjusted for gender, age, marital status, and education (Model 2). Finally, above that to establish whether the positive relationship between negative coping and recollected trauma and attachment insecurity are not only a spurious effect of general anxiety and/or depression we assessed the third group (Model 3) adjusted also for background levels of depression and general anxiety to compare groups of people already showing general negativism. Each of the independent variables was assessed in a separate model. All analyses were performed using the statistical software package IBM SPSS version 21 (IBM Corp., Armonk, NY, USA).

## 3. Results

### 3.1. Description of the Population

The background characteristics of the sample (mean age 51.1; SD = 17.2; 43.5% men) are presented in Table 1. Of all respondents 23.7% reported NRC score. Elderly respondents scored higher in NRC than younger respondents (*p* = 0.012). However, a comparison of the groups according religious practice (member of church, attending church services, prayer frequency) did not reveal any significant differences.

### 3.2. Negative Religious Coping and Experience in a Close Relationship

Table 2 shows the results of binary logistic regression aimed at assessing the associations of adult attachment (anxiety and avoidance) with NRC. The results of a crude and adjusted models were slightly different; in most cases the figures in model 3 (adjusted for general anxiety and depression) were lower than in model 1 (the crude one) and model 2 (adjusted only for sociodemographic variables). Item NRC-7 was significant only for anxiety in a close relationship adjusted for sociodemographic variables, however, after controlling for general anxiety and depression this association was not found. Both anxiety and avoidance in close relationships were associated with a significantly higher summary NRC, with an odds ratio (OR) = 1.27 (95% confidence interval (CI) 1.01–1.59) for anxiety and OR = 1.41 (1.13–1.75) for avoidance) after controlling for sociodemographic and general anxiety and depression variables.

### 3.3. Negative Religious Coping and Childhood Trauma Experience

The results of binary logistic regression assessing the associations of childhood trauma with negative religious coping and its separate items, crude and adjusted (Models 1–3) are presented in Table 3. The results obtained from regression models showed that each of the CTQ subscales was associated with higher NRC even after controlling for the spurious effect of general anxiety and depression. Physical neglect was associated with the highest risk of NRC with OR = 1.50 (1.23–1.83). Moreover, physical neglect was associated with a higher risk of NRC in each item separately. Physical neglect was also the only sub-scale that showed a significant association with the statement ‘I wonder whether God had abandoned me’ (NRC item 1).

## 4. Discussion

The aim of this study was to assess the associations of adult attachment and childhood trauma with negative religious coping. We found that almost a quarter of religious population showed signs of NRC and we also observed higher NRC within the group of elderly respondents. Furthermore, we found that NRC was associated with both anxiety and avoidance in close relationship and with all five types of childhood trauma experience.

The finding of higher NRC within the group of the elders is in line with results of other studies e.g., [45] and might be explained by usage of more active forms of coping among the young. The elders are, due to higher demands of active forms of coping and increased physiological vulnerabilities, more likely to use passive forms such as religious coping [46]. 

We also found that respondents who reported anxiety in adult relationships were more likely to report higher NRC. These findings are consistent with those of other studies [19,35]. An explanation could be that when individuals worry about whether their partner is available and reliable, they can transmit their feelings to God and thus use NRC strategies more often. Therefore, we could expect that although individuals with high attachment anxiety may seek help from God or their religious community [37], they might find these sources inadequate. However, the cross-sectional design of this study does not allow us to draw any conclusions on the direction of causality. They may be a mutual influence, as Fitchett [32] and Gall [47] stressed the possibility that a negative perception of God is associated to increased levels of anxiety and distress. Therefore, one’s views of God may affect relationship with the other people and a problematic attachment to them. Moreover, it is also possible that individuals with NRC might be less likely to experience a safe relationship to God or to their religious community, which may consequently strengthen their insecure attachment style. Moreover, these participants might further feel abandoned or punished by God as a projection of their personal attachment style [19].

Additionally, we found that attachment avoidance was associated with NRC, which corresponds to the findings of Schottenbauer et al. [20], who reported attachment avoidance qualities as a predictor of NRC. However, our results diverge from Pollard et al. [19], who found no interaction between NRC and attachment avoidance. An explanation for this difference could be that the respondents who reported high attachment avoidance do not apply NRC strategies in a consistent way [19], therefore, the results in various studies might vary. Our findings might be supported by the idea that attachment anxiety and avoidance can be seen as a continuous state of insecurity [37] which could be distressing and may represent a negative impact on individual’s life. In a continuous state of distress or in a long-term exposure to negative events, NRC strategies are used more frequently [6], thus positive association between avoidance and NRC can occur. Our results consequently seem to support the correspondence theory, which suggests that for insecurely attached individuals, their relationship to God corresponds to their human relationships [17,35]. Individuals can therefore also transfer their human relationship difficulties to their relationship with God. 

Furthermore, we found that all subscales of the CTQ were associated with NRC. These results are consistent with the findings of other studies which have reported a negative impact of childhood trauma on religiosity [26,27,28]. Verbal, physical, and sexual mistreatment are related to difficulties in one’s attachment to God and may lead to a tendency to view God as less loving, and more distant and controlling [26]. Moreover, when CTQ subscales were assessed in their association with individual NRC items, physical neglect was found to be associated with each NRC item. Surprisingly, physical neglect was also the only subscale associated with the item focusing on abandonment by God. Thus, these results are contrasting to Granqvist’s compensational theory [48], that individuals who experienced a difficult childhood may develop a positive relationship with a higher power which would serve as a substitute and provide a secure base, so they do not feel abandoned. Moreover, as respondents reported also other forms of NRC (i.e., feeling punished or questioning God’s love and power) associated with childhood mistreatment, this rather supports the corresponding model [17] where children neglected by their parents may more often transmit their feelings to God and feel neither God cares for them and punish them.

In addition, we found no significant association between emotional and sexual abuse and some NRC items. Although respondents wondered what they had done that God would punish them, questioned God’s love, or felt abandoned by the religious community, they did not feel abandoned or punished by God for their lack of devotion. These findings contrast to those of other authors, who found strong associations between sexual and physical mistreatment and a concept of God as distant [27] and an association of feelings of distance from God with emotional neglect [49]. Nevertheless, as our respondents reported that childhood sexual abuse played no role in feeling abandoned by God, our results are consistent with a concept of God as a protective factor and a source of more positive forms of coping [27,28]. However, in these cases, the identity of the abuser seems to play an important role in the further perception of the trauma [27] and therefore should be considered in surveys while assessing the consequences for an individual’s relationship to God [48] and the tendency to use NRC strategies. The other explanation could be a social desirability bias in the survey that reflects the effort to report religious coping strategies in accordance with social expectations where negative attitudes to God could be considered morally unacceptable [50].

Finally, the comparison of the three models showed the differences between crude and adjusted data. Whereas the difference between crude model and model adjusted for age, gender, marital status and education was only slight, comparing these models to the model adjusted also for background levels of general anxiety and depression revealed differences. After checking for a spurious effect of general negativism in Model 3, the results showed no associations between anxiety in close relationship and NRC items except for the feelings of punishment from God and NRC summary. The comparison of groups in this model showed that association between NRC and childhood trauma and attachment avoidance and anxiety can be related to general negativism. Moreover, it is possible that adverse childhood experiences and attachment insecurity can be associated with higher adult anxiety and/or depression in general, which can consequently negatively influence one’s religious coping.

Our findings of an association between the feelings of being punished by God and negative religious coping support the idea of Pollard [19] that insecure attached individuals can feel punished by God as a projection of their attachment style. Moreover, Model 3 revealed similar results for associations between childhood trauma and negative religious coping. We found associations between physical neglect and all NRC items. This seems to be in line with the findings of other authors [17,27], that difficulties and experience of neglect in childhood may be reflected in the later perception of God and thus lead to increased usage of negative religious coping strategies.

### 4.1. Strengths and Limitations

This study has several important strengths. The most important is its response rate. It is also one of the few studies that assesses the associations of negative religious coping with adult attachment and childhood trauma experience in a secular environment. However, the high rate of religiously unaffiliated respondents in the original sample limited the sample size for this study. Another limitation is the cross-sectional design of the study, which does not allow us to make causal inferences. The third limitation may involve cultural awareness, as our study does not reflect a particular cultural context. Furthermore, the last limitation concerns information bias, as our data were based on self-reports of respondents, which might be influenced by social desirability as religiously affiliated respondents might have responded according to their images of God and religiosity. These limitations should be included in a follow-up study in order to achieve a better and more precise understanding of underlying processes that affect the tendency to use maladaptive religious coping.

### 4.2. Implications

Our findings suggest that attachment avoidance and anxiety as well as childhood experience of maltreatment may affect NRC. Framed within a multidisciplinary approach toward dealing with the history of childhood trauma or with the attachment insecurity, NRC might be worth considering for professional counselling interventions in the area of spirituality aimed at lowering the use of NRC. The counsellor or spiritual guide can obtain information about patient’s religious background or whether the patient uses religion to cope with his or her trauma. This can contribute to the culturally sensitive awareness of a counsellor.

At the same time, using NRC strategies can serve as a sign of attachment insecurity and distress, and could therefore be informative for professionals in other areas. Further research is needed to explore the role of religiosity in both one’s partner and one’s parents in the development of individual religiosity and one’s image of God. The role of a perpetrator of violence should be further considered. Moreover, further research should focus on unravelling the causal pathways.

## 5. Conclusions

Our findings suggest that adult attachment and childhood trauma are associated with negative religious coping. Attachment anxiety and avoidance may be transmitted to the relationship to God and lead to increased use of NRC strategies. Similarly, individuals who suffered any form of childhood trauma may tend to view God as rather distant and unloving, and they might be more likely to use NRC. Thus, this study offers a deeper understanding of the factors that might contribute to the use of maladaptive NRC.

## Figures and Tables

**Table 1:** Description of the study population, total and by religiosity/spirituality (R/S).

	Total		NRC ^a^		*p*-Value
	N	%	N	%	
**Gender**					
Male	231	43.5	56	24.2	n.s.
Female	300	56.5	70	23.3	
**Age**					
15–29 years old	86	16.2	13	16.0	
30–49 years old	154	29.0	30	19.5	0.013 *
50–69 years old	209	39.4	55	26.3	(1–4 *; 2–4 *)
70+ years old	82	15.4	28	34.1	
**Marital status**					
Single/Divorced/Widow-widower	199	37.5	49	24.6	n.s.
Married/Partner relationship	332	62.5	77	23.2	
**Education**					
Primary	37	7.0	11	29.7	
Skilled operative	150	28.2	37	24.7	n.s.
High school	234	44.1	56	23.9	
College	110	20.7	22	20.0	
**Religiosity** ^b^					
Believer, member of the church	170	32.0	44	25.9	n.s.
Believer outside the church	361	68.0	82	22.7	
**Church attendance**					
Attending	105	19.8	28	26.7	n.s.
Non-attending	426	80:2	98	23.0	
**Prayer**					
Praying regularly	131	24.7	37	28.2	n.s.
Not praying regularly	400	75.3	89	22.3	
**Total**	531	100	126	23.7	

Notes: ^a^ (NRC ≥ ‘quite a bit’ in any of the items); ^b^ independently of a church attendance; n.s. = non-significant; * *p* < 0.05. The *p*-value stands for comparison of all groups; results in parentheses show multiple group comparison with Bonferroni correction.

**Table 2:** Associations of experience in a close relationship (avoidance and anxiety) with negative religious coping and its items standardized to z-scores, crude, adjusted for age, gender, marital status, and education plus adjusted for raw anxiety and depression: results of binary logistic regression models leading to odds ratios with 95% confidence intervals.

	**NRC Summary**	**NRC-1** *Wondered whether God had abandoned me*	**NRC-2** *Felt punished by God for my lack of devotion*	**NRC-3** *Wondered what I did for God to punish me*
**Model 1 ^a^**				
Anxiety	1.42 (1.17–1.72) ***	1.35 (1.03–1.79) *	1.65 (1.24–2.18) **	1.61 (1.27–2.05) ***
Avoidance	1.47 (1.12–1.78) ***	1.44 (1.09–1.89) *	1.75 (1.32–2.32) ***	1.56 (1.23–1.99) ***
**Model 2 ^b^**				
Anxiety	1.50 (1.23–1.84) ***	1.42 (1.06–1.89) *	1.91 (1.42–2.56) ***	1.70 (1.33–2.18) ***
Avoidance	1.55 (1.27–1.91) ***	1.36 (1.01–1.85) *	1.85 (1.33–2.57) ***	1.75 (1.34–2.30) ***
**Model 3 ^c^**				
Anxiety	1.27 (1.01–1.59) *	1.20 (0.87–1.66)	1.64 (1.18–2.29) **	1.37 (1.03–1.82) *
Avoidance	1.41 (1.13–1.75) **	1.22 (0.88–1.70)	1.75 (1.23–2.48) **	1.53 (1.15–2.05) **
	**NRC-4** *Questioned God’s love for me*	**NRC-5** *Wondered whether my church had abandoned me*	**NRC-6** *Decided the devil made this happen*	**NRC-7** *Questioned* *the power of God*
**Model 1 ^a^**				
Anxiety	1.75 (1.31–2.25) ***	1.47 (1.09–1.99) *	1.65 (1.20–2.29) **	1.29 (0.94–1.77)
Avoidance	1.86 (1.42–2.44)***	1.92 (1.43–2.58) ***	2.23 (1.61–3.08) ***	1.23 (0.89–1.69)
**Model 2 ^b^**				
Anxiety	1.83 (1.39–2.42) ***	1.64 (1.21–2.23) **	1.42 (1.06–1.89) *	1.43 (1.03–1.97) *
Avoidance	2.02 (1.46–2.79) ***	2.24 (1.56–3.22) ***	3.53 (2.23–5.59) ***	1.24 (0.88–1.75)
**Model 3 ^c^**				
Anxiety	1.32 (0.95–1.84)	1.07 (0.73–1.57)	0.94 (0.61–1.45)	1.22 (0.84–1.76)
Avoidance	1.67 (1.17–2.37) **	1.87 (1.25–2.79) **	3.33 (1.98–5.61) ***	1.12 (1.77–1.63)

Notes: NRC–Negative Religious Coping; * *p* < 0.05, ** *p* < 0.01, *** *p* < 0.001. ^a^ Crude; ^b^ Adjusted for age, gender, marital status and education; ^c^ Adjusted for age, gender, marital status and education plus raw anxiety and depression may have a footer.

**Table 3:** Associations of childhood trauma experience with negative religious coping (summary and items) standardized to z-scores, crude, adjusted for age, gender, marital status, and education plus adjusted for raw anxiety and depression: results of binary logistic regression models leading to odds ratios (OR) with 95% confidence intervals (95% CI).

	**NRC Summary**	**NRC-1** *Wondered Whether God Had Abandoned Me*	**NRC-2** *Felt Punished by God for My Lack of Devotion*	**NRC-3** *Wondered What I Did for God to Punish Me*
**Model 1 ^a^**				
Emotional abuse	1.24 (1.03–1.49) *	0.90 (0.64–1.26)	1.14 (0.85–1.51)	1.37 (1.10–1.70) **
Physical abuse	1.33 (1.12–1.60) **	0.97 (0.68–1.31)	1.31 (1.04–1.66) *	1.41 (1.15–1.72) **
Sexual abuse	1.24 (1.04–1.47) *	1.12 (0.88–1.43)	1.20 (0.96–1.50)	1.20 (0.98–1.46)
Emotional neglect	1.48 (1.22–1.79) ***	1.35 (1.02–1.79) *	1.82 (1.30–2.42) ***	1.75 (1.38–2.23) ***
Physical neglect	1.72 (1.42–2.09) ***	1.57 (1.22–2.04) **	1.88 (1.44–2.44) ***	1.84 (1.46–2.31) ***
**Model 2 ^b^**				
Emotional abuse	1.30 (1.07–1.58) **	0.91 (0.64–1.29)	1.19 (0.87–1.62)	1.46 (1.15–1.86) **
Physical abuse	1.38 (1.13–1.66) **	0.91 (0.62–1.34)	1.35 (1.03–1.78) *	1.51 (1.20–1.90) ***
Sexual abuse	1.31 (1.09–1.58) **	1.19 (0.85–1.62)	1.30 (0.98–1.73)	1.29 (1.01–1.64) *
Emotional neglect	1.50 (1.22–1.87) ***	1.36 (0.99–1.86)	1.94 (1.41–2.66) ***	1.91 (1.45–2.51) ***
Physical neglect	1.65 (1.36–1.99) ***	1.49 (1.15–1.92) **	1.83 (1.40–2.39) ***	1.86 (1.48–2.34) ***
**Model 3 ^c^**				
Emotional abuse	1.12 (0.90–1.40)	0.74 (0.50–1.09)	0.91 (0.63–1.31)	1.20 (0.91–1.59)
Physical abuse	1.29 (1.05–1.59) *	0.85 (0.57–1.25)	1.25 (0.94–1.67)	1.41 (1.12–1.79) **
Sexual abuse	1.23 (0.99–1.54)	1.11 (0.81–1.52)	1.20 (0.90–1.62)	1.17 (0.91–1.50)
Emotional neglect	1.28 (1.01–1.62) *	1.13 (0.79–1.61)	1.68 (1.17–2.41) **	1.56 (1.15–2.12) **
Physical neglect	1.50 (1.23–1.83) ***	1.35 (1.02–1.78) *	1.65 (1.24–2.20) **	1.66 (1.30–1.12) ***
	**NRC-4** *Questioned God’s love for me*	**NRC-5** *Wondered whether my church had abandoned me*	**NRC-6** *Decided the devil made this happen*	**NRC-7** *Questioned* *the power of God*
**Model 1 ^a^**				
Emotional abuse	1.37 (1.07–1.75) *	1.36 (1.04–1.77) *	1.10 (0.78–1.54)	1.34 (1.02–1.76) *
Physical abuse	1.31 (1.05–1.65) *	1.31 (1.03–1.68) *	1.46 (1.15–1.87) **	1.34 (1.05–1.7) *
Sexual abuse	1.36 (1.13–1.65) **	1.51 (1.26–1.82) ***	1.41 (1.56–1.73) **	1.22 (0.97–1.54)
Emotional neglect	1.80 (1.38–2.36) ***	1.94 (1.44–2.61) ***	1.95 (1.42–2.68) ***	1.58 (1.17–2.14) **
Physical neglect	1.85 (1.44–2.39) ***	2.36 (1.77–3.13) ***	2.08 (1.55–2.80) ***	1.69 (1.28–2.24) ***
**Model 2 ^b^**				
Emotional abuse	1.49 (1.14–1.94) **	1.43 (1.07–1.91) *	1.10 (0.76–1.58)	1.36 (1.02–1.83) *
Physical abuse	1.39 (1.07–1.81) *	1.37 (1.04–1.82) *	1.51 (1.15–1.98) **	1.36 (1.03–1.80) *
Sexual abuse	1.55 (1.22–1.98) ***	1.74 (1.36–2.24) ***	1.53 (1.19–1.97) **	1.30 (0.97–1.75)
Emotional neglect	1.91 (1.40–2.59) ***	2.12 (1.52–2.97) ***	1.88 (1.35–2.62) ***	1.60 (1.14–2.25) **
Physical neglect	1.81 (1.40–2.35) ***	2.34 (1.76–3.12) ***	2.07 (1.45–2.95) ***	1.58 (1.20–2.10) **
**Model 3 ^c^**				
Emotional abuse	1.12 (0.80–1.55)	0.96 (0.65–1.41)	0.58 (0.37–0.92) *	1.19 (0.86–1.65)
Physical abuse	1.29 (0.97–1.70)	1.24(0.91–1.69)	1.38 (1.03–1.86) *	1.28 (0.95–1.71)
Sexual abuse	1.39 (1.08–1.78) *	1.58 (1.22–2.03) ***	1.35 (1.02–1.77) *	1.23 (0.91–1.66)
Emotional neglect	1.37 (0.96–1.96)	1.47 (0.99–2.20)	1.35 (0.88–2.10)	1.41 (0.96–2.07)
Physical neglect	1.52 (1.14–2.02) **	2.04 (1.49–2.79) ***	1.74 (1.25–2.43) **	1.45 (1.07–1.96) *

Notes: NRC-negative religious coping; * *p* < 0.05, ** *p* < 0.01, *** *p* < 0.001. ^a^ Crude; ^b^ Adjusted for age, gender, marital status and education; ^c^ Adjusted for age, gender, marital status and education plus raw anxiety and depression.

## References

[1] Pargament K.I., Koenig H.G., Perez L.M. (2000). The many methods of religious coping: Development and initial validation of the RCOPE. J. Clin. Psychol..

[2] Pargament K.I., Smith B.W., Koenig H.G., Perez L. (1998). Patterns of positive and negative religious coping with major life stressors. J. Sci. Study Relig..

[3] Pargament K.I., Feuille M., Burdzy D. (2011). The Brief RCOPE: Current psychometric status of a short measure of religious coping. Religions.

[4] Ironson G., Kremer H., Lucette A. (2016). Relationship between spiritual coping and survival in patients with HIV. J. Gen. Intern. Med..

[5] Pargament K.I., Koenig H.G., Tarakeshwar N., Hahn J. (2004). Religious Coping Methods as Predictors of Psychological, Physical and Spiritual Outcomes among Medically Ill Elderly Patients: A Two-year Longitudinal Study. J. Health Psychol..

[6] Bjorck J.P., Thurman J.W. (2007). Negative life events, patterns of positive and negative religious coping and psychological functioning. J. Sci. Study Relig..

[7] Tarakeshwar N., Wanderwerker L.C., Paulk E., Pearce M.J., Kasl S.V., Prigerson H.G. (2006). Religious coping is associated with the quality of life of patients with advanced cancer. J. Palliat. Med..

[8] Herbert R., Zdaniuk B., Schulz R., Scheier M. (2009). Positive and negative religious coping and well-being in women with breast cancer. J. Palliat. Med..

[9] Latzer Y., Weinberger-Litman S., Gerson B., Rosch A., Mischel R., Hinden T., Kilstein J., Silver J. (2014). Negative religious coping predicts disordered eating pathology among Orthodox Jewish adolescent girls. J. Relig. Health.

[10] Paika V., Andreoulakis E., Ntountoulaki E., Papaioannou D., Kotsis K., Siafaka V., Fountoulakis K.N., Pargament K.I., Carvalho A.F., Hyphantis T. (2017). The Greek-Orthodox version of the Brief Religious Coping (B-RCOPE) instrument: Psychometric properties in three samples and associations with mental disorders, suicidality, illness perceptions, and quality of life. Ann. Gen. Psychiatry.

[11] Kharameh T.Z., Zamanian H., Montazeri A., Asgarian A., Esbiri R. (2016). Negative religious coping, positive religious coping, and quality of life among hemodialysis patients. Nephro-Urol. Mon..

[12] Ghorbani N., Watson P.J., Tahbaz S., Chen Z.J. (2017). Religious and psychological implications of positive and negative religious coping in Iran. J. Rel. Health.

[13] Rosmarin D.H., Pargament K.I., Flannelly K.J. (2009). Do spiritual struggles predict poorer physical/mental health among Jews?. Int. J. Psychol. Relig..

[14] Currier J.M., Smith P.N., Kuhlman S. (2017). Assessing the unique role of religious coping in suicidal behavior among US Iraq and Afghanistan veterans. Psychol. Relig. Spiritual..

[15] Pargament K.I., Koenig H.G., Tarakeshwar N., Hahn J. (2001). Religious struggle as a predictor of mortality among medically ill elderly patients: A 2-year longitudinal study. Arch. Inter. Med..

[16] Kirkpatrick L.A., Shaver P.R. (1992). An attachment-theoretical approach to romantic love and religious belief. Pers. Soc. Psychol. Bull..

[17] Rowatt W.C., Kirkpatrick L.A. (2002). Two dimensions of attachment to God and their relation to affect, religiosity, and personality constructs. J. Sci. Study Relig..

[18] Granqvist P., Hagekull B. (1999). Religiousness and perceived childhood attachment: Profiling socialized correspondence and emotional compensation. J. Sci. Study Relig..

[19] Pollard S.E., Riggs S.A., Hook J.N. (2014). Mutual influences in adult romantic attachment, religious coping, and marital adjustment. J. Fam. Psychol..

[20] Schottenbauer M.A., Klimes-Dougan B., Rodriguez B.F., Arnkoff D.B., Glass C.R., LaSalle V.H. (2006). Attachment and affective resolution following a stressful event: General and religious coping as possible mediators. Ment. Health Relig. Cult..

[21] Hvidtjørn D., Hjelmborg J., Skytthe A., Christensen K., Hvidt N.C. (2014). Religiousness and religious coping in a secular society: The gender perspective. J. Relig. Health.

[22] Lawrence R.T. (1997). Measuring the Image of God: The God Image Inventory and the God Image Scales. J. Psychol. Theol..

[23] Counted V. (2015). Understanding God images and God concepts: Towards a pastoral hermeneutics of the God attachment experience. Verbum Eccles..

[24] Noffke J.L., McFadden S.H. (2001). Denominational and Age Comparisons of God Concepts. J. Sci. Study Relig..

[25] Granqvist P. (2002). Attachment and religiosity in adolescence: Cross-sectional and longitudal evaluations. Pers. Soc. Psychol. Bull..

[26] Reinert D.F., Edwards C.E. (2009). Attachment theory, childhood maltreatment, and religiosity. Psychol. Relig. Spiritual..

[27] Bierman A. (2005). The effects of childhood maltreatment on adult religiosity and spirituality: Rejecting God the Father because of abusive fathers?. J. Sci. Study Relig..

[28] Gall T.L., Basque V., Damasceno-Scott M., Vardy G. (2007). Spirituality and the current adjustment of adult survivors of childhood sexual abuse. J. Sci. Study Relig..

[29] Tedeschi R.G., Calhoun L.G. (2004). Posttraumatic growth: Conceptual foundations and empirical evidence. Psychol. Inq..

[30] Grossoehme D.H., Fitchett G. (2013). Testing the validity of a protocol to screen for spiritual struggle among parents of children with cystic fibrosis. Res. Social. Sci. Study Relig..

[31] Ironson G., Stuetzle R., Fletcher M.A. (2006). An increase in religiousness/spirituality occurs after HIV diagnosis and predicts slower disease progression over 4 years in people with HIV. J. Gen. Inter. Med..

[32] Fitchett G., Murphy P.E., Kim J., Gibbons J.L., Cameron J.R., Davis J.A. (2004). Religious struggle: Prevalence, correlates and mental health risks in diabetic, congestive heart failure, and oncology patients. Int. J. Psychiatry Med..

[33] Fitchett G., Risk J.L. (2009). Screening for spiritual struggle. J. Pastoral Care Counsel..

[34] Thune-Boyle I.C., Stygall J., Keshtgar M.R.S., Davidson T.I., Newman S.P. (2012). Religious/spiritual coping resources and their relationship with adjustment in patients newly diagnosed with breast cancer in the UK. Psychooncology.

[35] Giordano A.L., Cashwell C.S., Lankford C., King K., Henson R.K. (2017). Collegiate sexual addiction: Exploring religious coping and attachment. J. Couns. Dev..

[36] Birgegard A., Granqvist P. (2004). The correspondence between attachment to parents and God: Three experiments using subliminal separation cues. Pers. Soc. Psychol. Bull..

[37] Granqvist P. (2005). Building a bridge between attachment and religious coping: Test of moderators and mediators. Ment. Health Relig. Cult..

[38] Granqvist P., Hagekull B. (2003). Longitudal Predictions of Religious Change in Adolescence: Contribution from the Interaction of Attachment and Relationship Status. J. Soc. Pers. Relatsh..

[39] Pew Research Center Global Religious Diversity: Half of the Most Religiously Diverse Countries are in Asia-Pacific Region. April 2014. http://www.pewresearch.org/wp-content/uploads/sites/7/2014/04/Religious-Diversity-full-report.pdf.

[40] Fraley R.C., Waller N.G., Brennan K.A. (2000). An item-response theory analysis of self-report measures of adult attachment. J. Pers. Soc. Psychol..

[41] Kascakova N., Husarová D., Hasto J., Kolarcik P., Solcová I.P., Gecková A.M., Tavel P. (2016). Validation of a 16-item short form of the Czech version of the Experiences in Close Relationships Revised Questionnaire in a representative sample. Psychol. Rep..

[42] Bernstein D.P., Stein J.A., Newcomb M.D., Walker E., Pogge D., Ahluvalia T., Zule W. (2003). Development and validation of a brief screening version of the Childhood Trauma Questionnaire. Child. Abuse Negl..

[43] Derogatis L.R., Melisaratos N. (1983). The Brief Symptom Inventory-An Introductory Report. Psychol. Med..

[44] Kabat J., Kascakova N., Furstova J., Bartuskova L., Glogar P., Polackova Solcova I., Tavel P. (2018). Psychometricke charakteristiky ceske verze Strucneho inventare priznaku (BSI-53). [Psychometric Characteristics of the Czech version of the Brief Syptom Inventory (BSI-53)]. Ceskoslov. Psychol..

[45] Noffke L., Hall T.W. (2007). Chapter 4. Attachment psychotherapy and God image. J. Spiritual. Ment. Health.

[46] Folkman S., Lazarus R.S., Pimley S., Novacek J. (1987). Age Differences in Stress and Coping Processes. Psychol. Aging.

[47] Gall T.L. (2004). Relationship with God and the quality of life of prostate cancer survivors. Qual. Life Res..

[48] Granqvist P. (1998). Religiousness and perceived childhood attachment: On the question of compensation or correspondence. J. Sci. Study Relig..

[49] Kennedy P., Drebing C.E. (2002). Abuse and religious experience: A study of religiously committed evangelical adults. Ment. Health Relig. Cult..

[50] Exline J.J., Kaplan K.J., Grubbs J.B. (2012). Anger, exit, and assertion: Do people see protest toward God as morally acceptable?. Psychol. Relig. Spiritual..

